# Lobectomy may not be suitable for patients with follicular neoplasm cytology

**DOI:** 10.3906/sag-1610-125

**Published:** 2020-02-13

**Authors:** Bekir UÇAN, Mustafa ŞAHİN, Mustafa ÖZBEK, Muhammed KIZILGÜL, Müyesser SAYKİ ARSLAN, Mustafa ÇALIŞKAN, Güleser SAYLAM, Erman ÇAKAL

**Affiliations:** 1 Department of Endocrinology and Metabolism, Dışkapı Yıldırım Beyazıt Training and Research Hospital, Ankara Turkey; 2 Department of Endocrinology and Metabolism, School of Medicine, Ankara University, Ankara Turkey; 3 Department of Otolaryngology and Head & Neck Surgery, Dışkapı Yıldırım Beyazıt Training and Research Hospital, Ankara Turkey

**Keywords:** Follicular neoplasm, lobectomy, thyroid carcinoma

## Abstract

**Background/aim:**

The most appropriate surgical management of follicular neoplasm/suspicious for follicular neoplasm (FN) lesions is still contradictory. We aimed to evaluate the data of our patients with follicular neoplasm treated with thyroidectomy.

**Materials and methods:**

We retrospectively analyzed the data of 74 patients who were diagnosed with follicular neoplasm cytology (FN cytology) by fine needle aspiration biopsy (FNAB) and had undergone total thyroidectomy or lobectomy with isthmectomy (LwI).

**Results:**

We examined a total of 74 patients, of which 64 (83.7%) were female and 10 (16.3%) were male. The malignancy rate in the pathological examinations of these patients was 31/74 (41.9%). The most common cancer among the patients with malignancy was papillary thyroid carcinomas (PTC) (20/31, 65%). Among the subtypes of PTCs, 11 were classical PTC, 5 were a follicular variant of PTC, 2 were the oncocytic variant of PTC, 1 was the diffuse sclerosing variant, and 1 was a columnar cell variant of PTC.

**Conclusion:**

Since most FN cytology has been pathologically diagnosed with papillary cancer and some papillary cancer subtypes have been unfavorable pathologically, total thyroidectomy should be the most suitable treatment option in this group. Lobectomy with LwI is not suitable for patients with FNAB-proven FN cytology.

## 1. Introduction 

The incidence of thyroid cancer is increasing around the world as a result of increased use of ultrasonography. Thyroid nodules were found in 20%–50% of the population, depending on both population and environmental factors (1,2). Fine needle aspiration biopsy (FNAB) is an inexpensive, minimally invasive outpatient procedure that determines the malignancy risk in thyroid nodules. The Bethesda classification for thyroid cytology is used for the pathologic classification of these nodules (3). This classification system subdivides the ‘indeterminate’ thyroid cytology in compliance with malignancy risk. About 5%–15% of cases called atypia or follicular lesion of undetermined signiﬁcance, 15%–30% suspected follicular neoplasm (FN) or Hürthle cell neoplasm (HCN) cases, and 60%–75% suspected malignancy cases prove to be malignant (4). FN and HCN are relatively uncommon diseases, and their cytologic diagnosis is difficult in comparison to papillary thyroid carcinoma (PTC), which poses a cytologic accuracy higher than 90%. The diagnostic accuracy and predictive malignancy rates of FN and HCN are less than those in PTC when a histologic correlation is performed (5). 

According to the American Thyroid Association Management Guidelines for Adult Patients with Thyroid Nodules and Differentiated Thyroid Cancer, surgery is recommended for patients when FN or HCN is diagnosed with FNAB, which cannot distinguish between benign and malignant tumors in these cases until the histologic diagnosis is performed from the surgery’ specimen (6). However, the most appropriate method of surgery for FN/follicular neoplasm-suspected lesions is still controversial. Lobectomy with isthmectomy (LwI) is the minimal therapeutic accepted procedure for patients with FN cytology, since FN has a malignancy risk lower than 30% and is usually unifocal. Malignancy rate was 19% in FN patients to whom LwI was applied, and this procedure was thought be adequate in 96% of patients with similar results to total thyroidectomy (TT) in terms of recurrence and survival (7,8).

Since multiglandular disease and thyroiditis may be associated with high malignancy risk, total thyroidectomy should be recommended in this case (9). PTC may show multifocal features and central lymph node metastasis at the time of diagnosis. Total thyroidectomy appears to be the most appropriate treatment option for patients with FN cytology, because most FN cytology was diagnosed with papillary carcinoma, and some have subtypes with poor prognosis.

We retrospectively evaluated the pathological correlation of FN cytology according to the Bethesda classification system in FNAB with the pathological findings in thyroidectomy specimens.  

## 2. Materials and methods

We retrospectively analyzed the data of 74 patients who were diagnosed with FN by FNAB and had undergone total thyroidectomy or lobectomy with isthmectomy between 2011 and 2013. A retrospective study was conducted at the Endocrinology Clinic of Dışkapı Yıldırım Beyazıt Teaching and Research Hospital, Ankara. The study was conducted in accordance with the Helsinki Declaration. Ethics committee approval and written informed consent of participants were obtained prior to the study. Patients with previous thyroid surgery were excluded. Ultrasonographic examinations were performed in our clinic with a 13-MHz linear transducer (EUB 7000 HV; Hitachi, Tokyo, Japan). Ultrasound (US) determined the largest and/or most suspicious-appearing thyroid nodule, defined as a dominant nodule. FNABs were performed with ultrasound guidance, using either a 22-gauge needle attached to a 5-mL disposable plastic syringe or an aspirator. Samples were stained with hematoxylin and eosin and examined by our Pathology Department. All FNAB slides were reviewed by our experienced cytopathologist and regrouped into four main categories: nondiagnostic, benign, indeterminate, or malignant, as previously reported. The indeterminate group included atypia of undetermined significance (AUS) or follicular lesion of undetermined significance (FLUS), FN or suspicious for FN, HCN, and suspicious for papillary thyroid cancer (PTC) (10). Only patients presenting with follicular neoplasm/suspicious for FN cytology were included in the study. All operations were performed in our hospital. Intraoperative frozen section examination (IFSE) was not performed for diagnosing thyroid cancer or for determining the extent of thyroidectomy, because our pathologists consider the usefulness of this procedure to be very limited in patients with FN.

### 2.1. Statistical analysis

Statistical analysis was performed using PASW 18 (SPSS, Chicago, IL, USA). Normality was tested with the Kolmogorov–Smirnov and Shapiro–Wilk W tests. Data were described as mean and standard deviation for continuous variables, and as actual numbers and percentages for categorical variables. The comparison between the groups was performed with independent sample t-test and Fisher’s exact test for proportions. Statistical significance was defined as P < 0.05.

## 3. Results

Of a total of 74 patients, 64 (83.7%) were female and 10 (16.3%) were male. Mean age was 46.3 ± 12.2 years. Malignancy rate was 31/74 (41.9%) in the pathological examinations of these patients. Among the patients with malignancy, the most common cancer was papillary thyroid carcinomas (PTC) (20/31, 65%). Only 8 (26%) were diagnosed with follicular thyroid carcinoma, 2 (6%) were diagnosed with Hürthle cell carcinoma, and 1 (3%) was diagnosed as a neoplasm of uncertain malignant potential. Among PTC subtypes, 11 were classical PTC, 5 were the follicular variant of PTC, 2 were the oncocytic variant of PTC, 1 was the diffuse sclerosing variant, and 1 was a columnar cell variant, PTC. 

## 4. Discussion

The most appropriate surgery method for FN/follicular neoplasm-suspected lesions is still controversial. Thyroid FNAB is very sensitive in distinguishing between benign and malignant nodules and has limited sensitivity for follicular lesions. In these patients, clinical and ultrasonographic features are inadequate in predicting the malignancy potential of the lesion. According to the Bethesda system for reporting thyroid cytopathology, the malignancy risk for categories III and IV is 5%–15% and 15%–30%, respectively (10). Antunes et al. observed that malignancy risk was 45.83% in patients with FN who underwent LwI. Excluding microcarcinomas, 33.33% of patients from category IV were diagnosed with malignancy (11). Several other studies have also found higher rates of malignancy in patients with FN. Baloch et al. found a malignancy risk of 31% in patients with FN who had undergone TT (12). In another study, by Kim et al., this rate was found to be 47.9% (13). In our study, the malignancy rate in patients with FN was 41.9%. 

Spanheimer et al. observed that 18.81% of patients who had undergone LwI for nodular disease required completion thyroidectomy for thyroid cancer. In the same study, 42.2% of patients were required to be on lifelong thyroid hormone replacement therapy, and LwI was considered as an insufficient surgical method (14). 

In a study by Antunes et al., 40.1% of patients who had undergone LwI for FN required completion thyroidectomy for thyroid cancer, and no functional benefit was achieved in 23.1% of these patients because of post-LwI hypothyroidism. Nodular relapse occurred in 20.35% of patients undergoing LwI. The authors recommended total thyroidectomy for patients with FN cytology preoperative TSH > 2.16 mU/L, and, in Bethesda category IV, younger than 39.5 years (11). In another study conducted in the last period, it was observed that 47% of patients with indeterminate cytology (Category III and Category IV) who underwent LwI became hypothyroid postoperatively (15). According to a meta-analysis, the risk of malignancy in 2571 patients with FN cytology was 26.1% (16). The high rate of malignancy in our patients with FN cytology supports the thesis that total thyroidectomy might be considered at the forefront.

Approximately 16% of the thyroid nodules evaluated by FNAB are determined to be follicular neoplasms that require excisional biopsy, since only histopathological evidence of capsular and vascular invasion can confirm malignancy (17,18). LwI is recommended as the minimum surgical procedure to be performed on FN or suspicious for FN (10). Several authors suggest the implementation of total thyroidectomy in these cases, because it eliminates the possibility of reoperation and the sonographic follow-up. In a recent study, the authors proposed that total thyroidectomy is the most cost-effective treatment option for follicular thyroid nodules (19).

The use of intraoperative frozen section examination (IFSE) during diagnostic LwI for an FN is contradictory. Since IFSE may allow intraoperative diagnosis of malignancy, it may prevent the patient from being reoperated for completion thyroidectomy. However, the sensitivity of IFSE is low and it rarely affects the course of the operation; instead, it adds to costs and extends operation times. Additionally, possible false positive results by IFSE may cause an unnecessary thyroid lobe removal on the contralateral side (20). Thyroid cancer was diagnosed in 26 (27%) patients, with a diagnosis of FN on findings from FNAB who underwent surgery (21). In the light of this information, LwI does not seem to be a viable surgery option due to the high risk of malignancy and possible failure of IFSE.

Columnar cell, tall cell, diffuse sclerosing variant types of PTC, and the widely invasive variant of follicular carcinoma have a worse prognosis. These variants are characterized by widely extrathyroidal invasion, vascular invasion, widely tumor necrosis, and a high grade of mitosis. Trabecular and insular carcinoma variants are other undifferentiated aggressive tumor histologies. (22). Since some of our patients have these subtypes of aggressive tumors, we think that total thyroidectomy would be beneficial in terms of mortality and morbidity.

In conclusion, LwI may not be suitable for patients with FN cytology because of the high malignancy risk in patients with FN cytology.
